# Psychological Factors, Physical Conditions, and Functioning Among US Veterans

**DOI:** 10.1001/jamanetworkopen.2024.27382

**Published:** 2024-08-09

**Authors:** Ian C. Fischer, Peter J. Na, David B. Feldman, Alex H. Krist, Harold S. Kudler, Dilip V. Jeste, Robert H. Pietrzak

**Affiliations:** 1 National Center for PTSD, Veterans Affairs (VA) Connecticut Healthcare System, West Haven; 2 Department of Psychiatry, Yale School of Medicine, New Haven, Connecticut; 3 Psychiatry Service, VA Connecticut Healthcare System, West Haven; 4 Department of Counseling Psychology, Santa Clara University, Santa Clara, California; 5 Department of Family Medicine and Population Health, Virginia Commonwealth University, Richmond; 6 Department of Psychiatry and Behavioral Sciences, Duke University, Durham, North Carolina; 7 Department of Veterans Affairs Mid-Atlantic Mental Illness Research, Education, and Clinical Center (VISN6 MIRECC), Durham, North Carolina; 8 Global Research Network on Social Determinants of Mental Health and Exposomics, La Jolla, California; 9 Department of Social and Behavioral Sciences, Yale School of Public Health, New Haven, Connecticut

## Abstract

This cross-sectional study examines the association of protective psychological characteristics with mental, psychosocial, cognitive, and physical functioning in military veterans.

## Introduction

The US Department of Veterans Affairs Whole Health initiative is designed to provide a holistic, personalized approach to health care that supports functioning and cultivates well-being.^1^ Military veterans experience a variety of stressors (eg, deployment) and health conditions (eg, cardiovascular disease)^2^ that can undermine functioning and erode well-being. Identification of factors associated with these domains may inform health promotion interventions in veterans.

Herein, we expanded previous work,^3^ which found that positive (ie, adaptive) psychological factors are associated with greater well-being in the presence of physical health difficulties among US veterans, to identify modifiable factors in multiple functional domains in this population. Although this study was largely exploratory, we hypothesized that positive psychological factors (eg, purpose in life) would moderate the association between risk factors (eg, health difficulties) and functioning.

## Methods

Data were obtained from the National Health and Resilience in Veterans Study (NHRVS), a nationally representative survey conducted between November 18, 2019, and March 8, 2020. Poststratification weights were used to yield results representative of the veteran population. The Human Subjects Committee of the VA Connecticut Healthcare System approved the study. Participants provided electronic informed consent. We followed the STROBE reporting guideline. The eMethods in Supplement 1 provides additional study details.

Past-month mental and physical functioning were self-reported by participants using the Short Form-8 Health Survey, 7 domains of psychosocial difficulties were assessed using the Brief Inventory of Psychosocial Functioning, and 6 domains of subjective cognitive functioning were assessed using the Medical Outcomes Study Cognitive Functioning Scale-Revised. The eTable in Supplement 1 provides descriptions of all measures. We assessed self-reported race and ethnicity to characterize the sample’s demographic composition and adjust for any implications of race and ethnicity in multivariable models.

Linear regression and relative importance analyses were conducted to identify unique correlates of scores on each functioning measure; only significant bivariate correlates were included in these models (Bonferroni-corrected *P* = .003). Planned post hoc analyses were conducted to identify individual items or scales comprising the strongest protective factor (eg, protective psychological characteristics) associated with each functioning measure (*P* < .001). Interaction terms of the strongest risk and protective factors were incorporated to test for moderation. Data analysis was performed in March 2024 using SPSS 28 (IBM) and R 4.3.3. (R Project for Statistical Computing).

## Results

Overall, 4069 veterans (3670 males [90.2%], 399 females [9.8%]; mean [SD] age, 62.2 [15.7] years) completed the NHRVS. The sample comprised individuals with Black (296 [11.2%]), Hispanic (307 [6.6%]), White (3318 [78.1%]), and mixed or other (148 [4.1%]) race and ethnicity.

**Figure 1.**
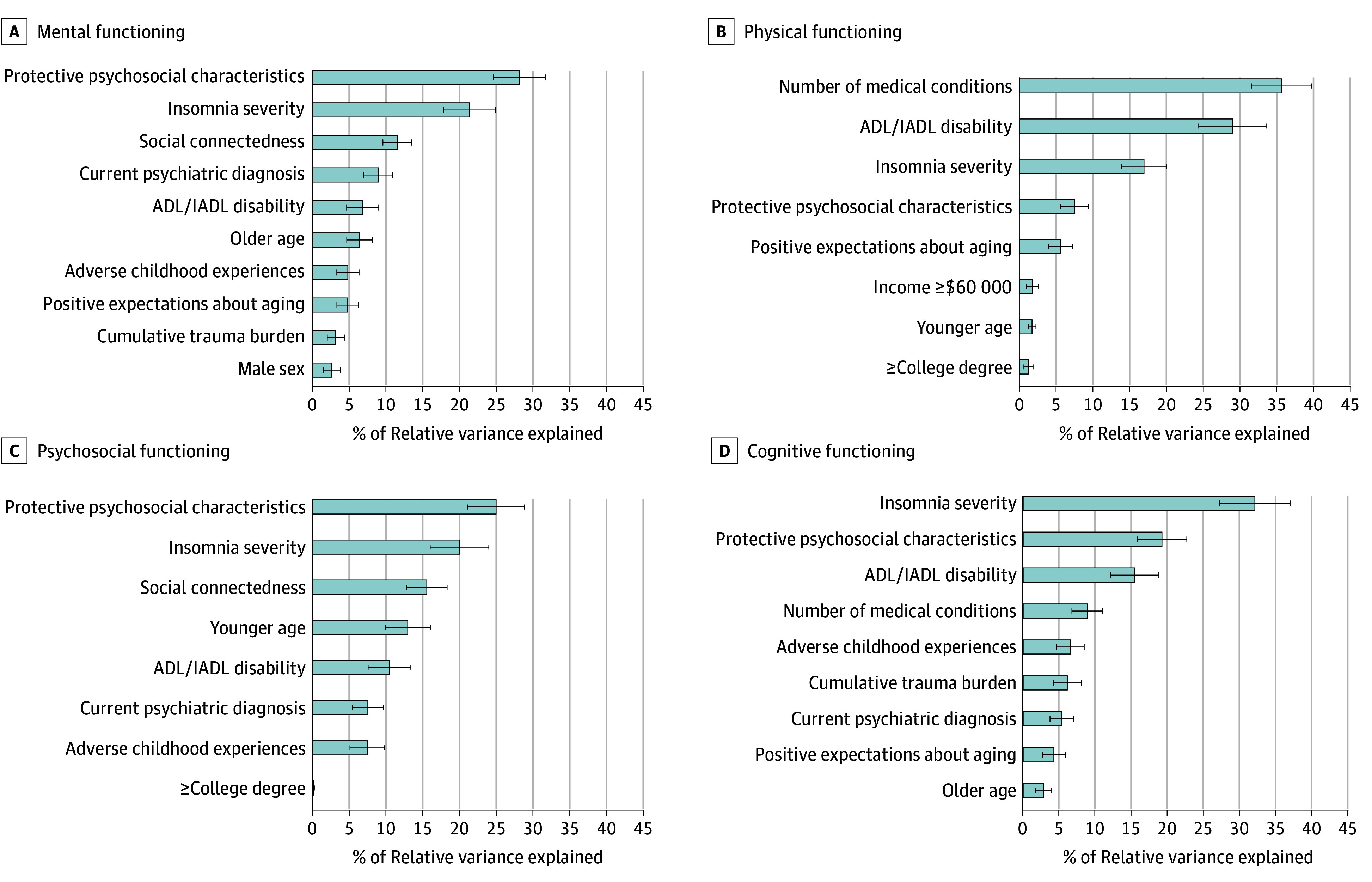
Relative Importance Analyses of Correlates of Subjective Mental, Physical, Psychosocial, and Cognitive Functioning ADL indicates activities of daily living; IADL, instrumental activities of daily living. Error bars indicate 95% CIs.

Composite scores of protective psychological characteristics (ie, resilience, purpose in life, gratitude, optimism, grit, curiosity, community integration) and insomnia severity explained the majority of variance in mental, psychosocial, and cognitive functioning scores. For physical functioning, number of medical conditions and disability in activities or instrumental activities of daily living explained the majority of variance (Figure 1).

**Figure 2.**
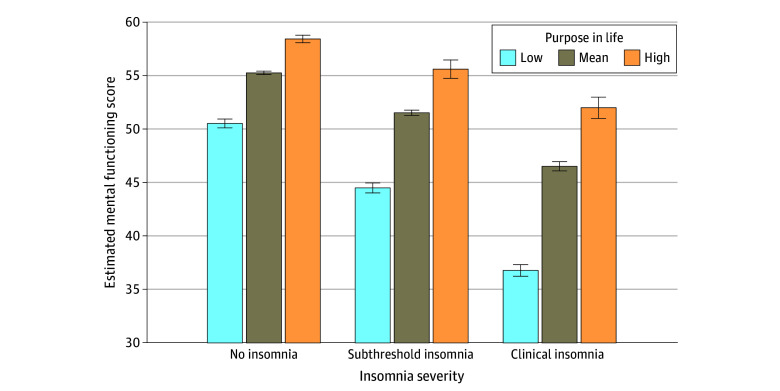
Estimated Mental Functioning Scores by Insomnia Severity and Purpose in Life Error bars indicate 95% CIs. Scores of 0 to 7 on the Insomnia Severity Index were indicative of no insomnia; 8 to 14, subthreshold insomnia; and 15 to 28, clinical insomnia. Low purpose in life represents scores less than or equal to 1 SD below the sample mean, whereas high purpose in life represents scores greater than or equal to 1 SD above the sample mean.

Planned post hoc and exploratory interaction analyses revealed that purpose in life (Figure 2), optimism, and grit moderated the association between insomnia severity and mental functioning. Among veterans with clinical insomnia, those with higher levels of each protective factor reported greater functioning. Similar interactions were observed for psychosocial and cognitive functioning.

## Discussion

This up-to-date analysis found that physical difficulties explained most variance in physical functioning, whereas a composite measure of positive psychological characteristics and insomnia severity explained most variance in mental, psychosocial, and cognitive functioning. Furthermore, positive psychological factors (eg, purpose in life) involved in goal-setting moderated the adverse associations of the strongest risk factor (ie, insomnia) with mental, psychosocial, and cognitive functioning.

While conventional medical care may be positioned to address physical functioning, identifying and treating insomnia^4^ and promoting valued goal pursuits^5^ may be needed to sustain overall functioning and well-being (ie, Whole Health) in veterans. Study limitations include the cross-sectional design and reliance on self-reported measures. Further research is needed to identify causal factors in multidomain functioning.

## Notes

Supplement 1.
eMethods.
**eTable.** Study Measures
eReferences

Supplement 2.
Data Sharing Statement
