# Supplementary material for: Psychological Factors, Physical Conditions, and Functioning Among US Veterans
Source: JAMA Netw Open. 2024 Aug 9;7(8):e2427382. doi: 10.1001/jamanetworkopen.2024.27382 (PMC11316230; doi:10.1001/jamanetworkopen.2024.27382)
Supplement: Supplement 1. — eMethods. eTable. Study Measures eReferences [file jamanetwopen-e2427382-s001.pdf]

Supplemental Online Content

Fischer IC, Na PJ, MD, Feldman DB, et al. Psychological factors, physical conditions, and functioning among US veterans. *JAMA Netw Open*. 2024;7(8):e2427382. doi:10.1001/jamanetworkopen.2024.27382

eMethods.

eTable. Study Measures

eReferences

This supplemental material has been provided by the authors to give readers additional information about their work.

© 2024 Fischer IC et al. *JAMA Network Open*

eMethods

Sample

The National Health and Resilience in Veterans Study (NHRVS) is a nationally representative survey of U.S. veterans that was conducted between 11/18/19 and 3/8/20 (median completion date: 11/21/2019). In total, 7,860 survey panelists were invited to participate in the NHRVS and 4,069 completed the survey (51.8% completion rate). Data regarding survey responders and non-responders were not available.

The NHRVS sample was drawn from KnowledgePanel, a research panel of more than 50,000 households that is maintained by Ipsos, a survey research firm. KnowledgePanel® is a probability-based, online non-volunteer access survey panel of a nationally representative sample of U.S. adults that covers approximately 98% of U.S. households. Panel members are recruited through national random samples, originally by telephone and now almost entirely by postal mail. Households are provided with access to the Internet and computer hardware if needed. KnowledgePanel® recruitment uses dual sampling frames that include both listed and unlisted telephone numbers, telephone and non-telephone households, and cell-phone-only households, as well as households with and without Internet access.

Demographic data of survey panel members are assessed regularly by Ipsos using the same set of questions used by the U.S. Census Bureau. Race/ethnicity was assessed via self-report using a standard set of questions used by the U.S. Census Bureau; this information was assessed in the current study to characterize the demographic composition of the sample and to adjust for any influence of race/ethnicity in multivariable models.

To permit generalizability of study results to the entire population of U.S. veterans, the Ipsos statistical team computed post-stratification weights using the following benchmark distributions of U.S. military veterans from the most recent (August 2019) Current Veteran Population Supplemental Survey of the U.S. Census Bureau's American Community Survey: age, gender, race/ethnicity, Census Region, metropolitan status, education, household income, branch of service, and years in service. An iterative proportional fitting (raking) procedure was used to produce the final post-stratification weights.

All participants provided informed consent and the study was approved by the Human Subjects Committee of the VA Connecticut Healthcare System.

The NHRVS followed the Strengthening the Reporting of Observational Studies in Epidemiology (STROBE) and American Association for Public Opinion Research (AAPOR) reporting guidelines for cohort studies.

© 2024 Fischer IC et al. *JAMA Network Open*

eTable. Study measures

**Variable**	**Assessment**
Functioning measures	
Mental health	Score on the Mental Component Summary, a composite of the four mental health items of the Short Form Health Survey-8.[1] Scores range from 0-100, with a score of 50 (SD=10) representing the average level of functioning in the general population. Higher scores indicate better functioning.
Physical health	Score on the Physical Component Summary, a composite of the four physical health items of the Short Form Health Survey-8.[1] Scores range from 0-100, with a score of 50 (SD=10) representing the average level of functioning in the general population. Higher scores indicate better functioning.
Psychosocial difficulties	Score on the Brief Inventory of Psychosocial Functioning (B-IPF).[2] Assesses past-month difficulties in seven domains (i.e., romantic relationships, family relationships, parenting, friendships and socializing, training/education, work, and self-care).
Cognition	Score on Medical Outcomes Study Cognitive Functioning Scale.[3] Assesses past-month difficulties in six domains (i.e., reasoning, concentration/thinking, confusion, memory, attention, and reaction time).
Sociodemographic characteristics	
	A general sociodemographic questionnaire was used to assess age, sex, race/ethnicity, education, marital status, status, and annual household income.
Psychiatric and health characteristics	
Adverse childhood experiences (ACEs)	Adverse Childhood Experiences Questionnaire[4] total score.
Cumulative trauma burden	Life Events Checklist for DSM-5 total score.[5]
Any current psychiatric disorder	Positive screen for a diagnosis of one or more of the following disorders: posttraumatic stress disorder (PTSD), Major depressive disorder (MDD), generalized anxiety disorder (GAD), alcohol use disorder (AUD), or drug use disorder (DUD). Positive screens were based on the following criteria: PTSD: Score $\geq$33 on the PTSD Checklist for DSM-5, past month;[6] MDD and GAD: Score $\geq$3 on the Patient Health Questionnaire for Anxiety and Depression (PHQ-4);[7] AUD: Score on alcohol use disorder module from the DSM-5 version of the Mini Neuropsychiatric Interview;[8] and DUD: Score on drug use disorder module from the DSM-5 version of the Mini Neuropsychiatric Interview.[8]
Insomnia severity	Score on Insomnia Severity Index.[9]

© 2024 Fischer IC et al. *JAMA Network Open*

Number of medical conditions	Sum of number of medical conditions endorsed in response to question: "Has a doctor or healthcare professional ever told you that you have any of the following medical conditions?" (e.g., arthritis, cancer, diabetes, heart disease, asthma, kidney disease). Range: 0-24 conditions.
ADL/IADL disability	Any disability in activities of daily living or instrumental activities of daily living. The following questions was asked: "At the present time, do you need help from another person to do the following?" (e.g., bathe; walk around your home or apartment; get in and out of chair). Endorsement of any of these activities was indicative of having a disability with an activity of daily living. Any disability in instrumental activities of daily living. The following question was asked: "At the present time, do you need help from another person to do the following?" (e.g., pay bills or manage money; prepare bills; get dressed). Endorsement of any of these activities was indicative of having a disability.[10]
Lifetime nicotine use disorder	Score ≥ 5 on the Fagerström Test for Nicotine Dependence (FTND) scale was considered a positive screen.[11]
Protective characteristics	
Protective psychological characteristics	A composite score of adaptive psychological traits[12,13] was used to assess dispositional attitudes and capacities for coping that are associated with more positive mental health outcomes, including qualities such as resilience; a sense of life purpose; dispositional gratitude, optimism, curiosity/exploration, grit; and perceived community integration. Resilience was measured using the Connor-Davidson Resilience Scale,[14] a 10-item scale with items such as "I am able to adapt when changes occur," measured on a scale from 1 ("not at all") to 5 ("nearly true all the time"); Cronbach's α=0.93. The Purpose in Life Test, Short Form,[15] a 4-item scale, was used to index sense of meaning and purposefulness in life, assessed on a scale from 1 ("no goals/purpose/progress/meaning") to 7 ("very clear goals/purpose/progress/meaning"; Cronbach's α=0.89), and the Short Grit Scale[16], an 8-item scale with items such as "I finish whatever I begin," measured on a scale from 1 ("Not at all like me") to 5("Very much like me") was used to assess grit; Cronbach's α=0.97. Dispositional gratitude, optimism, and curiosity were each assessed using single 7-point Likert scale items adapted from the Gratitude Questionnaire (GQ-6)[17]; the Life Orientation Test-Revised (LOTS-R)[18]; and the Curiosity and Exploration Inventory-II (CEI-II)[19], respectively. Sense of community integration and acceptance was assessed with a single item, "I feel well integrated in my community."
Positive expectations regarding aging	Sum score of the following 3 items from the Expectations Regarding Aging scale[20]: "Every year that people age, their energy levels go down a little more;" "It is normal to be depressed when you are old;" and "Forgetfulness is a natural occurrence just from growing old."

© 2024 Fischer IC et al. *JAMA Network Open*

Social connectedness	Composite score of responses to questions assessing structural social support (prompt “About how many close friends and relatives do you have (people you feel at ease with and can talk to about what is on your mind)?”, perceived social support (Score on 5-item version of the Medical Outcomes Study Social Support Scale[21]), and attachment style (Endorsement of secure attachment (response a) to the following question: “Please select the statement below that best describes your feelings and attitudes in relationships[22]: (a) feeling that it is easy to get close to others and feeling comfortable with them (secure); (b) feeling uncomfortable being close to others (avoidant); or (c) feeling that others are reluctant to get close (anxious/ambivalent).

© 2024 Fischer IC et al. *JAMA Network Open*

eReferences

1. Stewart AL, Ware JE, Ware Jr JE. *Measuring functioning and well-being: the medical outcomes study approach*. duke university Press; 1992.
2. Marx B, Schnurr P, Lunney C, Weathers F, Bovin M, Keane T. The brief inventory of psychosocial functioning (B-IPF). *Retrived from: https://wwwptsdvagov/professional/assessment/documents/B-IPFpdf*. 2019;
3. Stewart AL, Ware J, Sherbourne CD, Wells KB. Psychological distress/well-being and cognitive functioning measures. *Measuring functioning and well-being: The medical outcomes study approach*. 1992:102-142.
4. Felitti VJ, Anda RF, Nordenberg D, et al. Relationship of childhood abuse and household dysfunction to many of the leading causes of death in adults. The Adverse Childhood Experiences (ACE) Study. *Am J Prev Med*. 1998;14(4):245-258.
5. Weathers F, Blake DD, Schnurr PP, Kaloupek DG, Marx BP, Keane TM. The Life Events Checklist for DSM-5 (LEC-5). Instrument available from the National Center for PTSD at www.ptsd.va.gov. Accessed July 13th, 2022.
6. Weathers FW, Litz BT, Keane TM, Palmieri PA, Marx BP, Schnurr PP. The PTSD Checklist for DSM-5 (PCL-5). *Scale available from the National Center for PTSD at wwwptsdvagov*. 2013;
7. Kroenke K, Spitzer RL, Williams JB, Löwe B. An ultra-brief screening scale for anxiety and depression: the PHQ–4. *Psychosomatics*. 2009;50(6):613-621.
8. Sheehan D. The mini-international neuropsychiatric interview, version 7.0 for DSM-5 (MINI 7.0). 2014;
9. Bastien CH, Vallières A, Morin CM. Validation of the Insomnia Severity Index as an outcome measure for insomnia research. *Sleep medicine*. 2001;2(4):297-307.
10. Hardy SE, Gill TM. Recovery from disability among community-dwelling older persons. *Jama*. 2004;291(13):1596-1602.
11. Heatherton TF, Kozlowski LT, Frecker RC, FAGERSTROM KO. The Fagerström test for nicotine dependence: a revision of the Fagerstrom Tolerance Questionnaire. *British journal of addiction*. 1991;86(9):1119-1127.
12. Pietrzak RH, Cook JM. Psychological resilience in older US veterans: Results from the national health and resilience in veterans study. *Depression and anxiety*. 2013;30(5):432-443.
13. Smith NB, Mota N, Tsai J, et al. Nature and determinants of suicidal ideation among US veterans: Results from the national health and resilience in veterans study. *Journal of Affective Disorders*. 2016;197:66-73.
14. Campbell-Sills L, Stein MB. Psychometric analysis and refinement of the connor–davidson resilience scale (CD-RISC): Validation of a 10-item measure of resilience. *Journal of Traumatic Stress: Official Publication of The International Society for Traumatic Stress Studies*. 2007;20(6):1019-1028.
15. Schulenberg SE, Schnetzer LW, Buchanan EM. The purpose in life test-short form: development and psychometric support. *Journal of Happiness Studies*. 2011;12(5):861-876.
16. Duckworth AL, Quinn PD. Development and validation of the Short Grit Scale (GRIT–S). *Journal of personality assessment*. 2009;91(2):166-174.
17. McCullough ME, Emmons RA, Tsang J. The grateful disposition: a conceptual and empirical topography. *J Pers Soc Psychol*. 2002;82:112-127.

© 2024 Fischer IC et al. *JAMA Network Open*

18. Glaesmer H, Rief W, Martin A, et al. Psychometric properties and population-based norms of the Life Orientation Test Revised (LOT-R). *British journal of health psychology*. 2012;17(2):432-445.
19. Kashdan TB, Gallagher MW, Silvia PJ, et al. The Curiosity and Exploration Inventory-II: Development, factor structure, and psychometrics. *J Res Pers*. 2009;43(6):987-998.
20. Sarkisian CA, Steers WN, Hays RD, Mangione CM. Development of the 12-item Expectations Regarding Aging Survey. *Gerontologist*. Apr 2005;45(2):240-8. doi:10.1093/geront/45.2.240
21. Sherbourne CD, Stewart AL. The MOS social support survey. *Social science & medicine*. 1991;32(6):705-714.
22. Hazan C, Shaver PR. Love and work: An attachment-theoretical perspective. *Journal of Personality and social Psychology*. 1990;59(2):270.

© 2024 Fischer IC et al. *JAMA Network Open*
