# Supplementary material for: Psychological Factors, Physical Conditions, and Functioning Among US Veterans
Source: JAMA Netw Open. 2024 Aug 9;7(8):e2427382. doi: 10.1001/jamanetworkopen.2024.27382 (PMC11316230; doi:10.1001/jamanetworkopen.2024.27382)
Supplement: Supplement 2. — Data Sharing Statement [file jamanetwopen-e2427382-s002.pdf]

Data Sharing Statement

Fischer. Psychological Factors, Physical Conditions, and Functioning Among US Veterans. *JAMA Netw Open*. Published August 09, 2024. doi:10.1001/jamanetworkopen.2024.27382

Data
Data available: No

Additional Information
Explanation for why data not available: Data available upon request.
